# Supplementary material for: The Effect of the Histone Chaperones HSPA8 and DEK on Tumor Immunity in Hepatocellular Carcinoma
Source: Int J Mol Sci. 2023 Jan 31;24(3):2653. doi: 10.3390/ijms24032653 (PMC9916749; doi:10.3390/ijms24032653)
Supplement: Supplementary file 1 [file ijms-24-02653-s001.zip › ijms-2076501-supplementary.pdf]

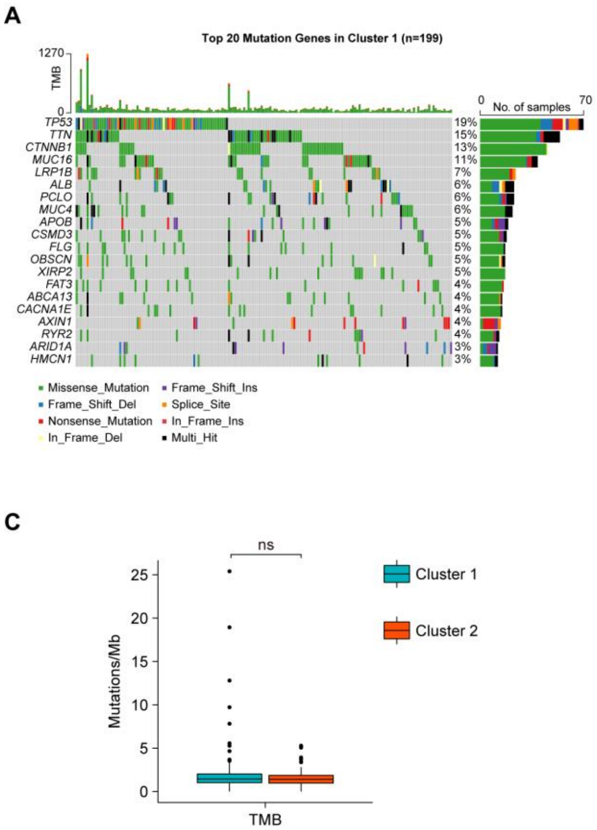

Supplementary Figure S1. Comparison of gene mutation profiles between the two clusters. Mutational landscape of Cluster 1 **(A)** and Cluster 2 **(B)**. **(C)** Comparison of TMB between the two clusters. ns, no significance.

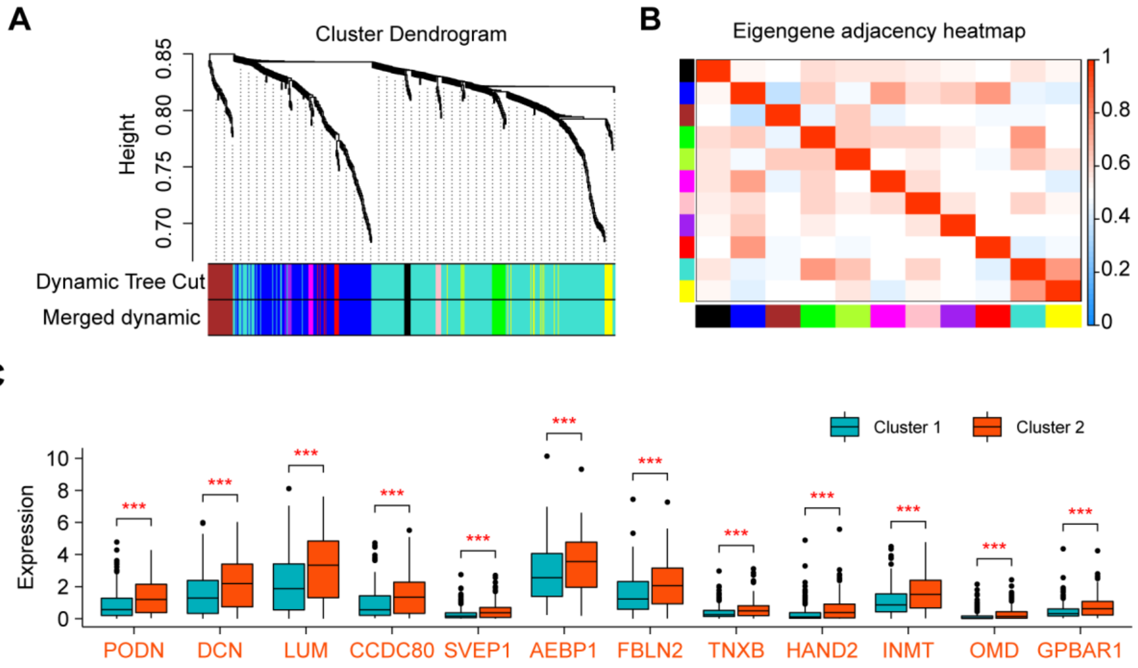

Supplementary Figure S2. Screening of hub genes related to immune infiltration by MGCNA. **(A)** Gene dendrogram and module colors. **(B)** Eigengene adjacency heatmap among the modules. **(C)** Comparison of the expression of the 12 hub genes between the two clusters.

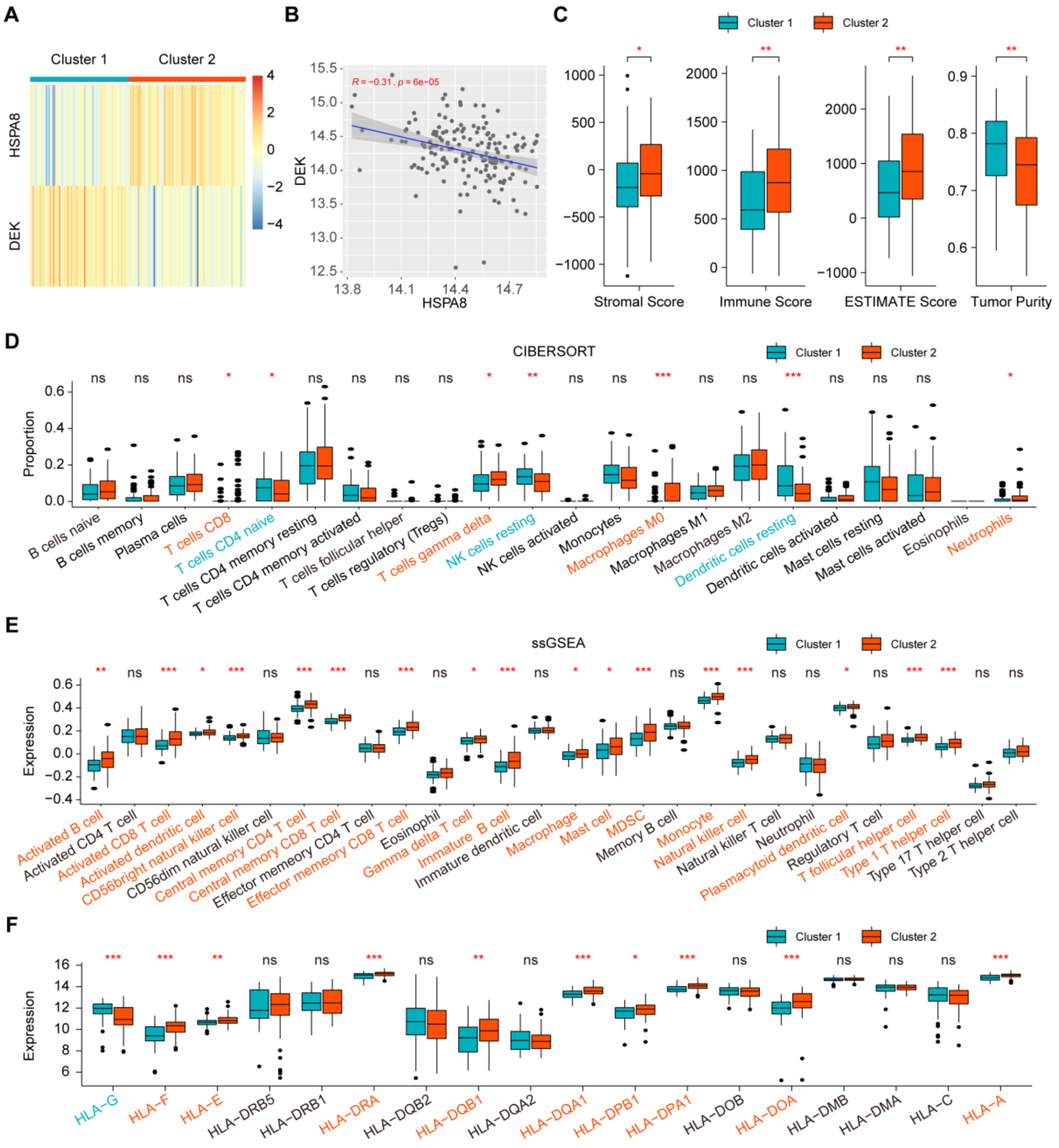

Supplementary Figure S3. Validation of immune infiltration of the two clusters by GSE19977. **(A)** GSE19977 patients are divided into two clusters according to *HSPA8* and *DEK*. **(B)** Association between the expression of *HSPA8* and *DEK*. **(C)** Comparison of the four indices of ESTIMATE. **(D)** Comparison of the proportion of immune cells by CIBERSORT. **(E)** Comparison of the expression of immune cells by ssGSEA. **(F)** Comparison of the expression of MHCs. ns, no significance, *P < 0.05, **P < 0.01, ***P < 0.001.

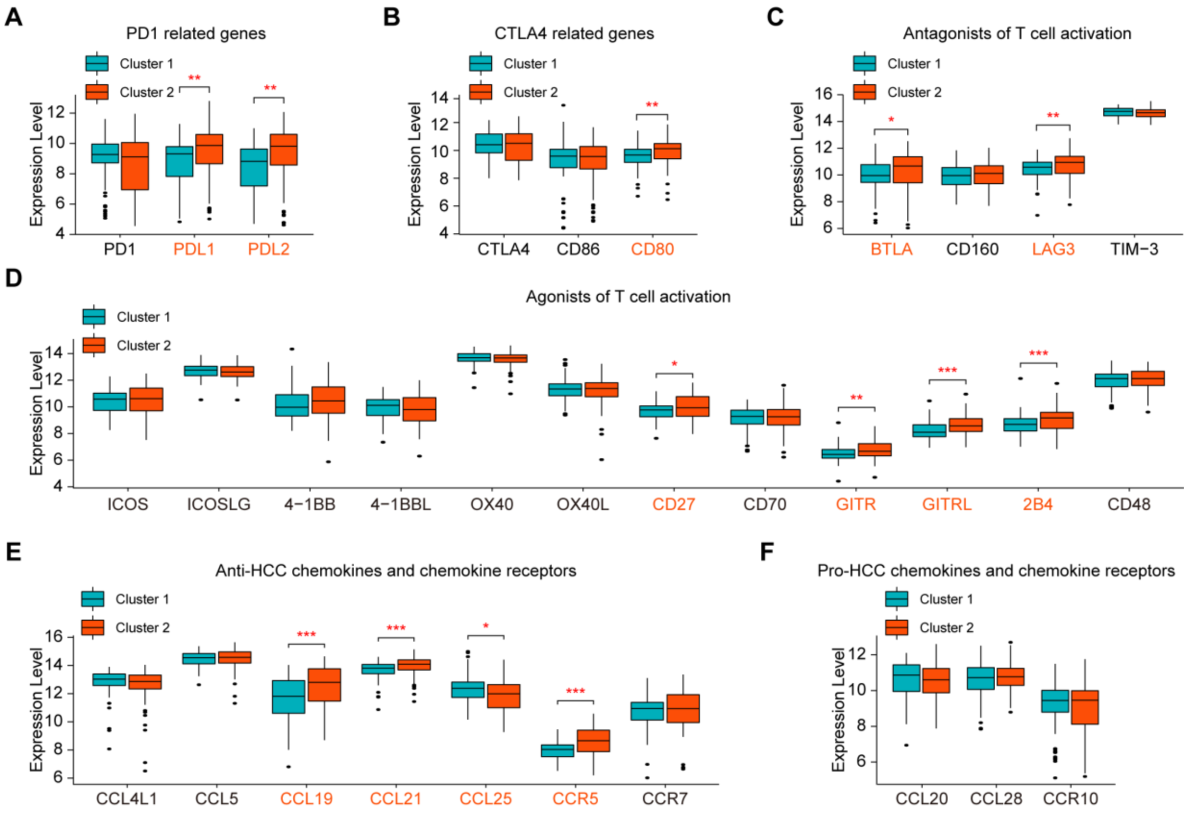

Supplementary Figure S4. Validation of sensitivity to immunotherapy of the two clusters by GSE19977. **(A-D)** Comparison of the expression of immunomodulatory genes. **(E, F)** Comparison of the expression of known chemokines and chemokine receptors that regulate the progression of HCC. *P < 0.05, **P < 0.01, ***P < 0.001.

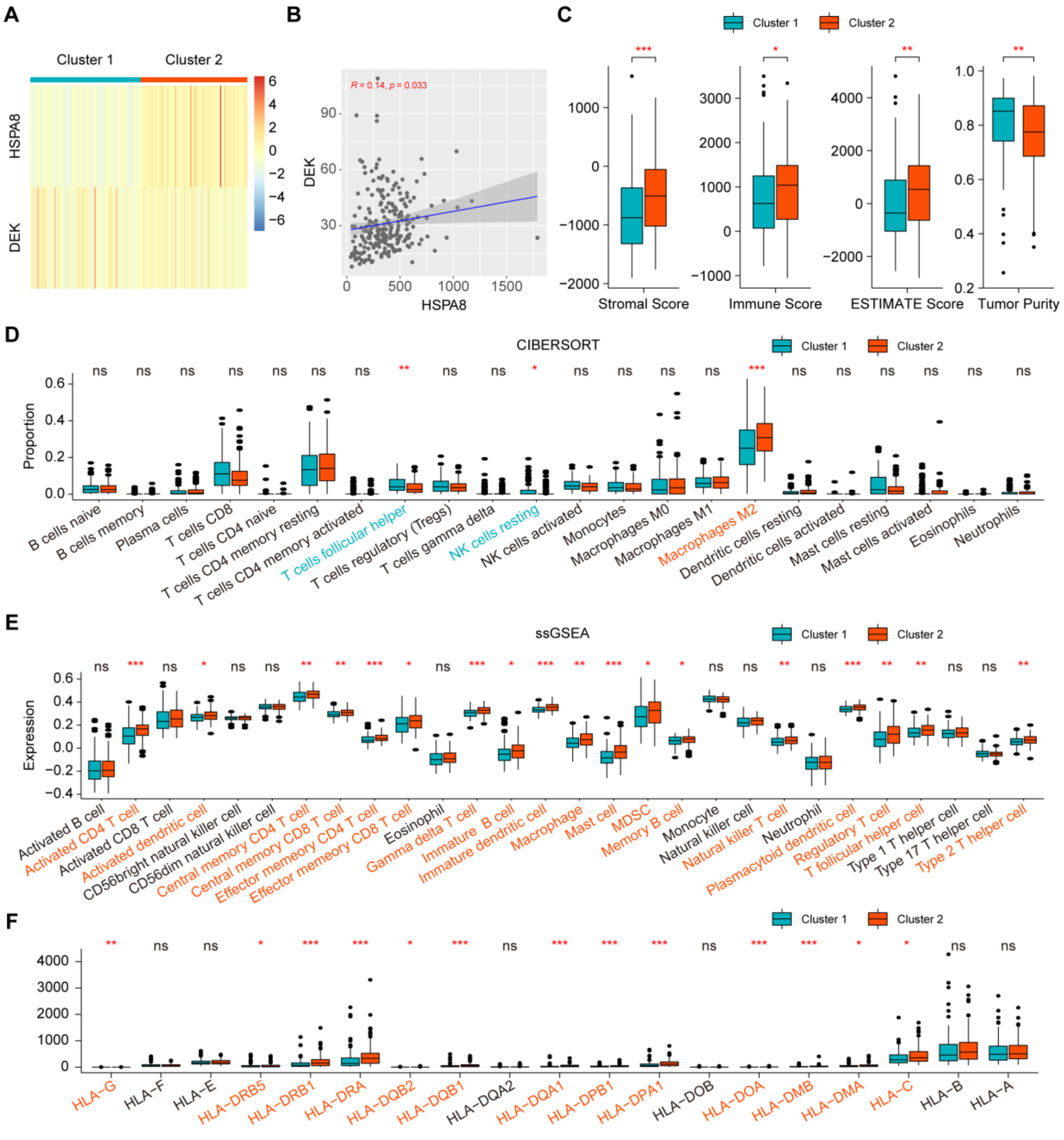

Supplementary Figure S5. Validation of immune infiltration of the two clusters by ICGC-LIRI JP. **(A)** ICGC-LIRI JP patients are divided into two clusters according to *HSPA8* and *DEK*. **(B)** Association between the expression of *HSPA8* and *DEK*. **(C)** Comparison of the four indices of ESTIMATE. **(D)** Comparison of the proportion of immune cells by CIBERSORT. **(E)** Comparison of the expression of immune cells by ssGSEA. **(F)** Comparison of the expression of MHCs. ns, no significance, *P < 0.05, **P < 0.01, ***P < 0.001.

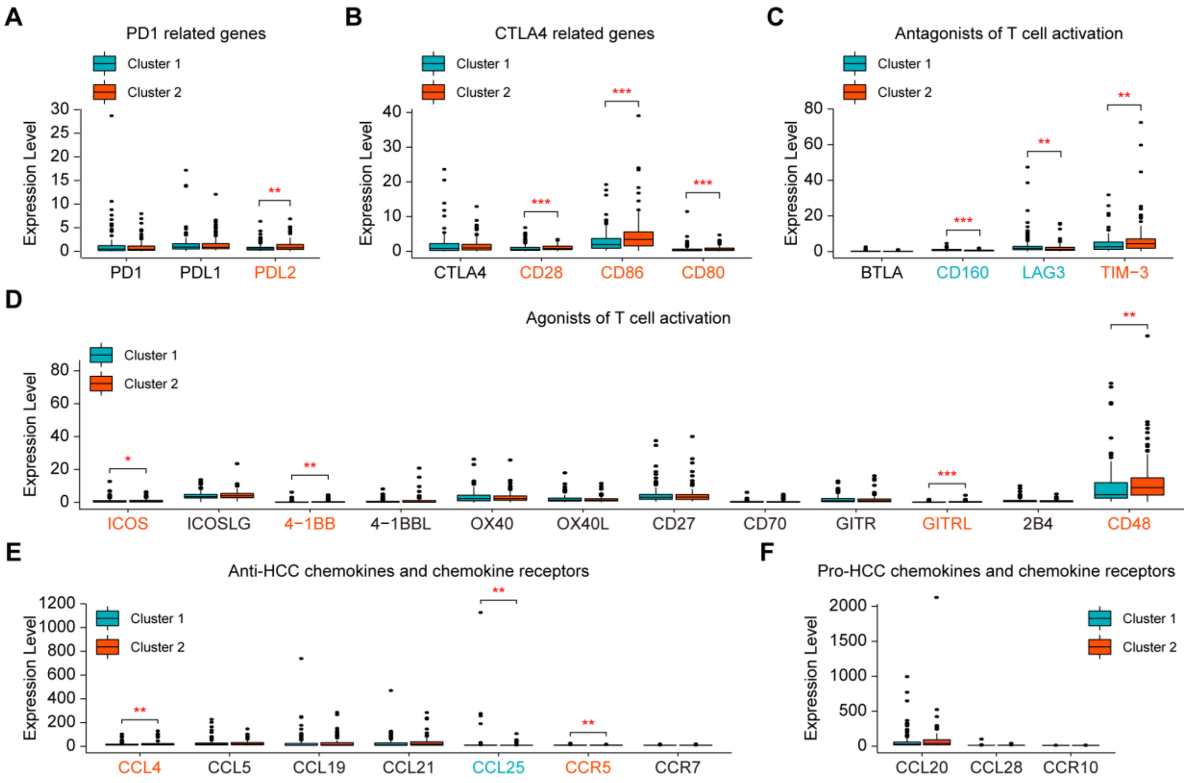

Supplementary Figure S6. Validation of immune infiltration of the two clusters by ICGC-LIRI JP. **(A-D)** Comparison of the expression of immunomodulatory genes. **(E, F)** Comparison of the expression of known chemokines and chemokine receptors that regulate the progression of HCC. *P < 0.05, **P < 0.01, ***P < 0.001.
